# Development and validation of a nomogram for predicting advanced liver fibrosis in patients with chronic hepatitis B

**DOI:** 10.3389/fmolb.2024.1452841

**Published:** 2024-09-02

**Authors:** Kexing Han, Jianfeng Wang, Xizhen Song, Luyang Kang, Junjie Lin, Qinggang Hu, Weijie Sun, Yufeng Gao

**Affiliations:** The First Affiliated Hospital of Anhui Medical University, Hefei, China

**Keywords:** chronic hepatitis B, liver fibrosis, nomogram, machine learning model, Lasso regression analysis

## Abstract

**Background:**

The progression of chronic hepatitis B (CHB) to liver fibrosis and even cirrhosis is often unknown to patients, but noninvasive markers capable of effectively identifying advanced liver fibrosis remains absent.

**Objective:**

Based on the results of liver biopsy, we aimed to construct a new nomogram to validate the stage of liver fibrosis in CHB patients by the basic information of CHB patients and routine laboratory tests.

**Methods:**

Patients with CHB diagnosed for the first time in the First Affiliated Hospital of Anhui Medical University from 2010 to 2018 were selected, and their basic information, laboratory tests and liver biopsy information were collected. Eventually, 974 patients were enrolled in the study, while all patients were randomized into a training cohort (n = 732) and an internal validation cohort (n = 242) according to a 3:1 ratio. In the training cohort, least absolute shrinkage and selection operator (Lasso) regression were used for predictor variable screening, and binary logistic regression analysis was used to build the diagnostic model, which was ultimately presented as a nomogram. The predictive accuracy of the nomograms was analyzed by running operating characteristic curve (ROC) to calculate area under curve (AUC), and the calibration was evaluated. Decision curve analysis (DCA) was used to determine patient benefit. In addition, we validated the built models with internal as well as external cohort (n = 771), respectively.

**Results:**

Ultimately, the training cohort, the internal validation cohort, and the external validation cohort contained sample sizes of 188, 53, and 149, respectively, for advanced liver fibrosis. Gender, albumin (Alb), globulin (Glb), platelets (PLT), alkaline phosphatase (AKP), glutamyl transpeptidase (GGT), and prothrombin time (PT) were screened as independent predictors. Compared with the aminotransferase-to-platelet ratio index (APRI), fibrosis-4 index (FIB-4), and King’s score, the model in the training cohort (AUC = 0.834, 95% CI 0.800–0.868, *p* < 0.05) and internal validation cohort (AUC = 0.804, 95% CI 0.742–0.866, *p* < 0.05) showed the best discrimination and the best predictive performance. In addition, DCA showed that the clinical benefit of the nomogram was superior to the APRI, FIB-4 and King’s scores in all cohorts.

**Conclusion:**

This study constructed a validated nomogram model with predictors screened from clinical variables which could be easily used for the diagnosis of advanced liver fibrosis in CHB patients.

## Introduction

Hepatitis B virus (HBV) infection is a global public health problem that puts enormous pressure on the global economy. Relevant studies have shown that globally an estimated 2.400 million people eventually turn into chronic hepatitis B (CHB) (Raptopoulou et al., 2009). The prognosis and treatment of CHB are closely correlated with the progression of liver fibrosis. China is a large country with HBV infection, and various degrees of liver fibrosis associated with CHB are the main cause of cirrhosis (Liang et al., 2015). Patients are often unaware of the progression of liver fibrosis, and the progression to decompensated liver disease will cause a series of acute and critical illnesses (Wu and Chang, 2015). Therefore, timely and accurate assessment of the degree of liver fibrosis is crucial to prevent CHB from progressing to cirrhosis, liver failure and hepatocellular carcinoma (D’Amico et al., 2006).

Liver biopsy is the gold standard for detecting liver fibrosis, but its invasiveness, high cost, and severe complications have limited its clinical use (Cadranel et al., 2000; Regev et al., 2002), and therefore have contributed to the development of noninvasive scoring tools for cirrhosis and liver fibrosis. Assessment of liver fibrosis with imaging means is a clinically important tool, but conventional methods such as ultrasound scanning, computed tomography and magnetic resonance imaging examinations still have limitations in assessing the presence or extent of liver fibrosis, in addition to being able to diagnose typical liver cirrhosis (Ding et al., 2021). Liver transient elastography (LUTE) for measuring liver stiffness has become an effective tool due to its high diagnostic performance. However, these studies have been conducted mainly in Western countries as well as applied mainly to patients with chronic hepatitis C (CHC), and the accuracy of the assessment of CHB-associated liver fibrosis still needs to be validated at this time (Jia et al., 2015). Due to the expensive equipment required for LUTE, it is still not fully available in primary care organizations in China. In addition, some other potential factors exist that may affect the accuracy of LUTE, such as excessive obesity, unexplained liver function injury, and operating technique errors (Wang et al., 2020).

Therefore, in recent years, the development of noninvasive tools which could response to the progression of liver fibrosis has become a hot research topic considering how to utilize the clinical characteristics of patients. For example, serum markers such as aspartate aminotransferase (AST) to platelet (PLT) ratio (APRI), fibrosis-4 score (FIB-4), and King’s score have been shown capable of assessing liver fibrosis (Imai et al., 2018; Wai et al., 2003; Ekin et al., 2022). However, these noninvasive scores were initially developed primarily for the assessment of patients with chronic hepatitis C. Their accuracy in assessing liver fibrosis staging remains controversial and has been repeatedly questioned in Chinese patient cohorts (Huang et al., 2019; Huang et al., 2015).

Nomogram is an effective model for visualizing regression equations (Kattan and Scardino, 2007), and has been widely used to predict the risk of development and prognostic patterns of many diseases (Huang et al., 2018; Wang X. et al., 2023; Dong et al., 2023). In addition, nomogram models are often applied to validate the effectiveness of previous scoring systems and to develop new ones. Nomograms need to be constructed with valid predictor variables. Therefore, it is crucial to screen for potential predictor variables. Among the many analytical methods, Lasso regression is a widely used regularized linear regression analysis method for variable selection, which enables variable selection and adjusts for complexity when fitting generalized linear models (Wen et al., 2024). In fact, the greatest advantage of Lasso regression is the ability to sparsify the predictor variables being screened. In this process, those unimportant predictor variables have a weight of 0 to the model as a whole. Screening of potential predictor variables minimizes the computation of eigenvalues and thus avoids overfitting (Daidone et al., 2024). Therefore, in this study, based on the advantages of Lasso regression analysis on potential predictor variables can be effectively screened. Using basic information and laboratory tests of patients with COPD, we aimed to develop and validate a new scoring system for the diagnosis of advanced liver fibrosis associated with COPD. We also compared the new score with other noninvasive models, including the APRI, FIB-4, and King scores.

## Methods

### Study patients

**FIGURE 1:**
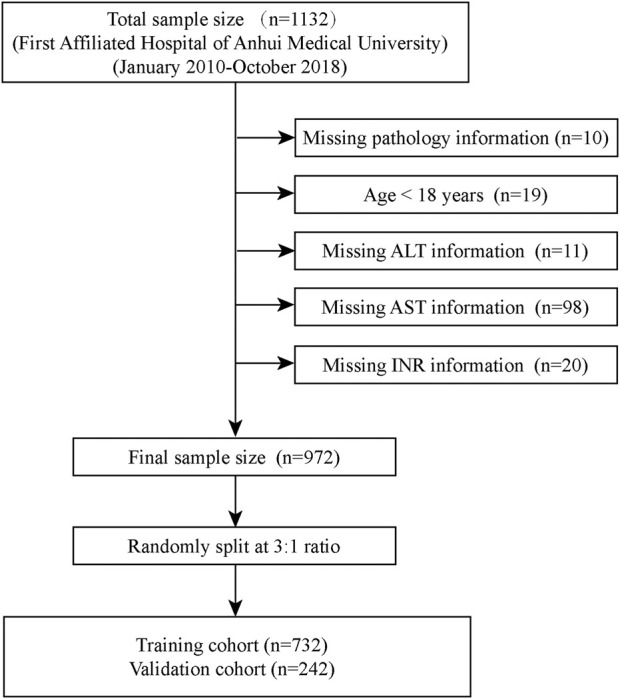
Flow chart for patients included in the training cohort. ALT, Alanine aminotransferase; AST, Aspartate aminotransferase; INR, International normalized ratio.

We retrospectively selected 1,132 patients with CHB initially attending the Department of Infectious Diseases of First Affiliated Hospital of Anhui Medical University from January 2010 to October 2018 for the study, all of whom had not been treated with antiviral therapy. We excluded 158 patients, and the specific screening process was shown in Figure 1. Meanwhile, we confirmed that the following diseases were excluded from the patients who were enrolled in the study: 1) obstructive jaundice caused by tumor compression, 2) previous liver transplantation, 3) drug-induced liver disease, 4) clinically significant portal hypertension, 5) the presence of hepatitis C virus, hepatitis D virus, autoimmune liver disease, alcoholic liver disease, and metabolic liver disease, 6) combined presence of extrahepatic fibrosis-related diseases such as connective tissue disease, chronic obstructive pulmonary disease, and interstitial pulmonary fibrosis. Finally, a total of 974 patients with CHB (hepatitis B surface antigen positive for more than 6 months) (Chinese Society of Infectious Diseases et al., 2019) were initially included in the study. Overall, we excluded participants who could not be used to calculate the noninvasive scores (FIB-4, APRI, King’s score) used to diagnose liver fibrosis. However, laboratory tests inevitably have missing values (Johansson and Karlsson, 2013). For this reason, we summarized the clinical characteristics of all patients to examine the pattern of missing values for each variable (Supplementary Table 1). Simply removing these missing values would result in a significant loss of information (Stavseth et al., 2019), and we populated the missing values accordingly. Continuous variables with a small number of missing values (<10%) were replaced with mean values, otherwise they were grouped according to a certain basis and the missing values were assigned to the missing group; categorical variables with a small number of missing values (n < 10) were directly excluded, otherwise the missing values were assigned to the missing group (Hao et al., 2022). Finally, all 974 patients were included in the study. Subsequently, we randomly split 732 patients as a training cohort for model development and 242 patients as an internal validation cohort based on a 3:1 ratio. Meanwhile, we retrospectively selected 771 patients from Anqing Municipal Hospital of Anhui Medical University as the external validation cohort. The study protocol was approved by the Ethics Committee of the First Affiliated Hospital of Anhui Medical College and followed the principles outlined in the Declaration of Helsinki. Given the retrospective nature of the study, the need for written informed consent from patients was waived.

### Clinical information collection

The basic information and clinical characteristics of each patient were obtained from the hospital’s electronic medical record. The basic information included age and gender. The clinical characteristics of the patients were mainly laboratory tests. For example, Alb, Glb, PLT, AKP, etc., and all laboratory tests were displayed in Supplementary Table 1. Blood samples for laboratory tests were obtained prior to liver tissue biopsy, and all patients were fasting when blood samples were obtained.

### Liver biopsy

Percutaneous liver biopsy was performed on the basis of ultrasound guidance and a 16G biopsy needle was used to collect the liver specimen during puncture. The criteria to be fulfilled for the obtained samples were a minimum length of not less than 1.5 cm and at least 7 complete confluent areas. The obtained liver samples were fixed in 10% formalin solution and subsequently embedded using paraffin and stained with hematoxylin-eosin (HE) (Zhang et al., 2021). All pathology sections were analyzed individually by two experienced pathologists and assured that they had no other additional information about the patient. The pathological sections of all patients were evaluated for the degree of liver fibrosis based on the Scheuer scoring system (Scheuer, 1991), which included S0 (no fibrosis), S1 (mild fibrosis without septa), S2 (moderate fibrosis with a small number of septa), S3 (severe fibrosis with a large number of septa but no cirrhosis) and S4 (cirrhosis). In this study, S3–S4 were defined as advanced fibrosis compared to S1–S2 (Ding et al., 2021).

### Formulas for existing noninvasive models

The formulas for APRI, FIB-4 and King’s score were as follows: APRI = AST (U/L)/upper limit of normal (set at 40 U/L) × 100/PLT (10^9^/L) (Wai et al., 2003); FIB-4 = [age (years) × AST (U/L)]/{LT (10^9^/L) × [ALT (U/L)]^1/2^} (Vallet-Pichard et al., 2007); King score = age (years) × AST (U/L) × International Normalized Ratio (INR)/PLT (10^9^/L) (Cross TJRizzi PBerry PABruce MPortmann BHarrison PM, 2009).

### Statistical analysis

Empower soft (http://www.empowerstats.com, X&Y Solutions, Inc., Boston, MA) and R software version 4.0.2 (http://www.r-project.org, Theon) were used for statistical analysis. Continuous variables were characterized by a normality test to determine their distribution and, if skewed, were expressed as median (interquartile range, IQR) and analyzed using the non-parametric Mann-Whitney U test. Categorical variables were expressed as rates or percentages and analyzed using the chi-square or Fisher exact test. In the training cohort, candidate predictors were screened using the Least Absolute Shrinkage and Selection Operator (LASSO) method to eliminate the potential effects of multicollinearity among variables, using Log(λ) = 1SE as the defining criterion for the screening variables and 10-fold cross-validation, where SE denotes the standard error of the λ value. The final predictors used to construct the model were determined from the candidate predictors based on multivariate logistic regression analysis. Using the rms software package we constructed a nomogram for the diagnosis of advanced liver fibrosis. Calibration curves were used to assess the agreement between predicted and observed values, and a receiver operating characteristic curve (ROC) was generated to evaluate the performance of the currently constructed nomogram and other noninvasive scores for the prediction of advanced liver fibrosis. Decision curve analyses were generated to assess the net benefit of our diagnostic model in several cohorts to demonstrate clinical utility. *p* < 0.05 was considered statistically different.

## Results

### Clinical characteristics of the patients

A total of 974 patients from the First Affiliated Hospital of Anhui Medical University were enrolled in the training cohort (732 patients) and the internal validation cohort (242 patients). The clinical characteristics of the patients in the training cohort and the internal validation cohort are demonstrated in Table 1. Based on the results of liver biopsy, the number of patients diagnosed as S3–S4 in the training cohort and the internal validation cohort were 188 (25.683%) and 23 (21.901%), respectively. Except for the lymphocyte count (LYC), the rest of the characteristics were not statistically significant between the two cohorts (*P* > 0.05). We obtained 771 cases from Anqing Municipal Hospital of Anhui Medical University as an external validation cohort. In Supplementary Table 2, the number of patients in the external validation cohort who were determined to be S3-S4 was 90 cases (11.673%). Between the training cohort and the external validation cohort, there were significant differences in all indicators except PLT, FIB-4, white cell count (WBC), red blood cell count (RBC), PLT, and LYC (*P* < 0.05).

**TABLE 1:** Clinical characteristics of studied patients in training cohort and internal validation cohort.

Characteristics	Training cohort	Internal validation cohort	*P*-value
Sample size	732	242	
Age (years)	37.000 (29.000–45.000)	36.500 (29.000–45.000)	0.861
Gender			0.354
Male	74.727	77.686	
Female	25.273	22.314	
AFP (ng/mL) (%)			0.815
<20	80.464	80.579	
≥20	7.240	6.198	
Unclear	12.295	13.223	
log _10_ (HBV DNA)	5.530 (3.867–7.250)	5.530 (4.062–7.195)	0.650
Alb (g/L)	41.050 (37.675–44.000)	40.650 (37.525–43.075)	0.139
Glb (g/L)	26.500 (23.500–29.600)	26.944 (24.300–29.675)	0.142
Tbil (umol/L)	13.600 (10.300–18.400)	14.850 (10.500–20.475)	0.122
Ibil (umol/L)	10.300 (8.100–14.700)	11.300 (8.525–16.500)	0.086
ALT (u/L)	47.000 (27.000–84.000)	48.500 (31.250–84.000)	0.548
AST (u/L)	34.000 (24.000–52.250)	33.000 (25.000–53.000)	0.805
AKP (u/L)	76.000 (61.000–98.000)	76.000 (61.000–99.750)	0.924
GGT (u/L)	26.000 (17.000–50.000)	27.500 (17.000–52.750)	0.610
TC (mmol/L) (%)			0.232
<5.2	73.634	73.554	
≥5.2	8.060	11.157	
Unclear	18.306	15.289	
TG (mmol/L) (%)			0.220
<1.7	69.809	69.008	
≥1.7	11.885	15.702	
Unclear	18.306	15.289	
WBC (10^9^/L)	5.165 (4.210–6.333)	5.300 (4.400–6.265)	0.370
NEU (10^9^/L)	2.725 (2.100–3.410)	2.800 (2.212–3.335)	0.595
LYC (10^9^/L)	1.795 (1.427–2.220)	1.886 (1.562–2.320)	0.011
RBC (10^12^/L)	4.600 (4.200–4.910)	4.685 (4.330–5.000)	0.189
Hb (g/L)	143.000 (130.000–152.000)	146.000 (133.000–153.000)	0.138
PLT (10^9^/L)	158.000 (116.000–198.000)	157.500 (118.250–188.000)	0.590
PT (s)	11.900 (11.100–13.000)	11.850 (11.000–13.075)	0.982
APTT (s)	31.200 (27.700–35.100)	31.857 (27.500–36.550)	0.170
INR	1.030 (0.970–1.110)	1.020 (0.962–1.090)	0.306
Liver fibrosis stage (%)			0.448
S0	3.142	5.372	
S1	49.044	50.826	
S2	22.131	21.901	
S3	12.978	11.570	
S4	12.705	10.331	
FIB-4	1.212 (0.781–1.993)	1.209 (0.753–2.021)	0.748
APRI	0.570 (0.357–1.039)	0.542 (0.375–1.097)	0.673
King’s score	8.552 (4.802–17.069)	8.482 (5.074–17.589)	0.738

AFP, alpha fetoprotein; AKP, alkaline phosphatase; HBV DNA, Hepatitis B virus deoxyribonucleic acid; APTT, activated partial thromboplastin time; Tbil, Total bilirubin; TC, total cholesterol; TG, triglyceride; NEU, neutrophil count; LYC, lymphocyte count; Ibil, Indirect bilirubin; GLb, Globulin; Alb, Albumin; ALT, alanine aminotransferase; AST, aspartate aminotransferase; GGT, glutamyl transpeptidase; WBC, white blood cell count; RBC, red blood cell count; Hb, hemoglobin; PLT, platelet count; PT, prothrombin time; INR, international normalized ratio; FIB-4, Fibrosis-4 score; APRI, aspartate aminotransferase to platelet ratio.

### Predictor variable selection and model development

**FIGURE 2:**
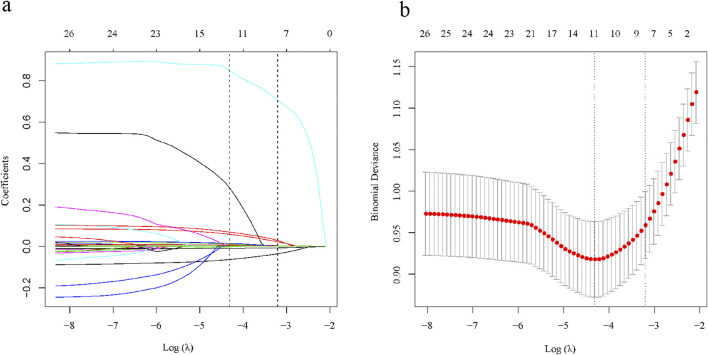
Selection of clinical characteristics based on LASSO regression. **(A)** Coefficient curves for 29 clinical characteristics; **(B)** LASSO regression and 10-fold cross-validation method to select the most appropriate clinical features.

There were a total of 29 potential variables for the clinical characteristics of all patients, and the Lasso regression analysis process was validated using the 10-fold crossover method, and a total of 7 parameters were extracted as candidate predictors [log(λ) = −3.204)], which were gender (male and female), ALB, GLB, PLT, AKP, GGT, and PT (Figure 2). Subsequently, we performed multivariate logistic regression analysis for all 8 candidate predictors to confirm that all included predictor variables met the criteria for model construction (Table 2). Based on the results of multivariate logistic regression analysis, we constructed a formula for advanced liver fibrosis and named it HBV-related advanced liver fibrosis index (HALF index), which was calculated as:

$$\begin{array}{ll} \text{HALF}\;\text{index} & =-0.433\;-\;0.804\times \left[\text{Gender}\;\left(\text{male}=1;\;\text{female}=0\right)\right]\\ & \;-\;0.093\times \text{ALB}+0.094\times \text{GLB}\;-\;0.012\times \text{PLT}\\ & \;+0.011\times \text{AKP}+0.005\times \text{GGT}+0.114\times \text{PT} \end{array}$$

**TABLE 2:** Results of multivariate logistic regression analysis for screened variables.

Variables	Estimate	Std error	OR	95% CI	*p*-value
Intercept	−0.433	1.183	0.648	0.064, 6.586	0.714
Alb (g/L)	−0.093	0.021	0.911	0.875, 0.949	<0.001
Glb (g/L)	0.094	0.021	1.098	1.055, 1.144	<0.001
PLT (10^9^/L)	−0.012	0.002	0.988	0.984, 0.992	<0.001
AKP (u/L)	0.011	0.004	1.011	1.004, 1.018	0.002
GGT (u/L)	0.005	0.002	1.005	1.001, 1.010	0.009
PT (s)	0.114	0.051	1.121	1.014, 1.238	0.026
Gender					
Male	Reference	Reference	Reference	Reference	Reference
Female	−0.804	0.263	0.447	0.267, 0.749	0.002

Alb, Albumin; GLb, Globulin; PLT, platelet count; AKP, alkaline phosphatase; GGT, glutamyl transpeptidase; PT, prothrombin time; OR, odds ratio; CI, 95% confidence interval.

### Construction and calibration of the nomogram

**FIGURE 3:**
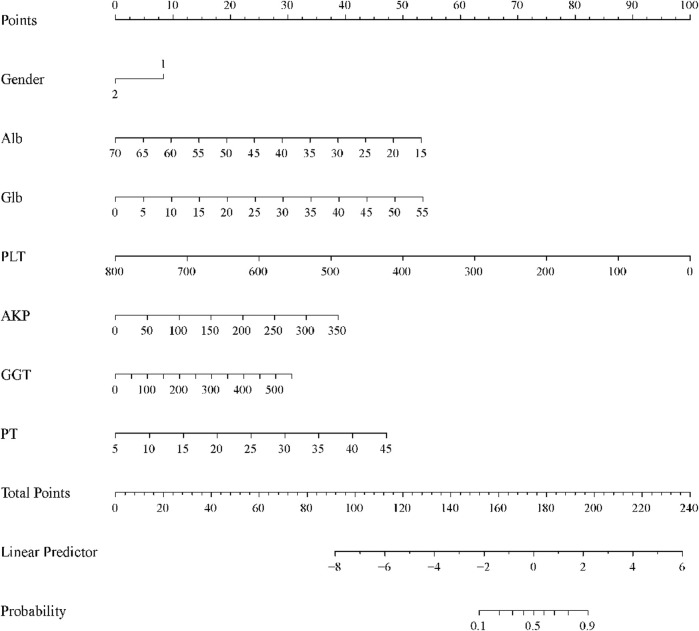
Nomogram for predicting the probability of CHB-associated advanced liver fibrosis. Each variable obtained a corresponding score on its respective scale, and the total score was the sum of the scores corresponding to the associated predictors, the total score obtained ultimately corresponding to the linear predictor. The probability of developing advanced liver fibrosis was determined based on the values corresponding to the linear predictors.

**FIGURE 4:**
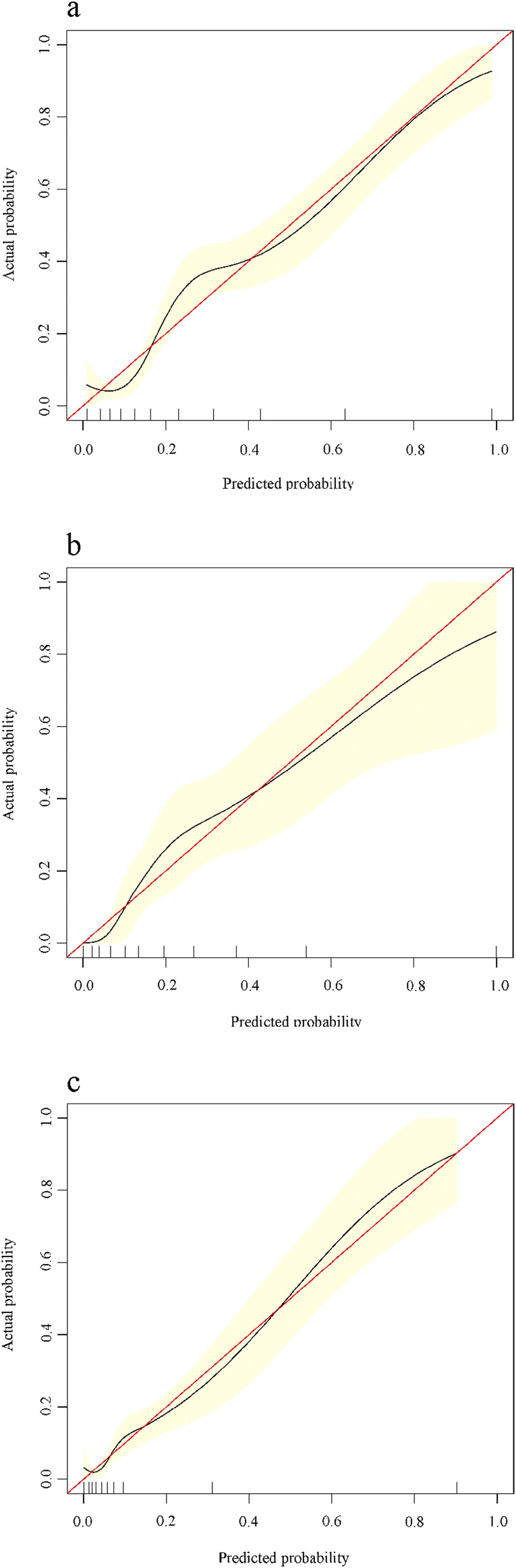
Calibration curves for predicting advanced liver fibrosis in patients with CHB. X-axis is the probability predicted by the nomogram, Y-axis is the actual probability. **(A)** Training cohort, **(B)** Internal validation cohort, **(C)** External validation cohort.

In the training cohort, based on the constructed predictive modeling formula, we constructed the nomogram for diagnosing advanced liver fibrosis containing all the screened independent predictors. The total score was the sum of the scores corresponding to the relevant predictors, and the obtained total score finally corresponded to the linear predictor. Ultimately, the values corresponding to the linear predictors were able to determine the probability of incident advanced liver fibrosis (Figure 3). In addition, in all cohorts, the results of the calibration curves showed that the predicted values of the HALF index exhibited good agreement with the actual observed values (Figure 4).

### Predictive performance and validation of nomogram

**FIGURE 5:**
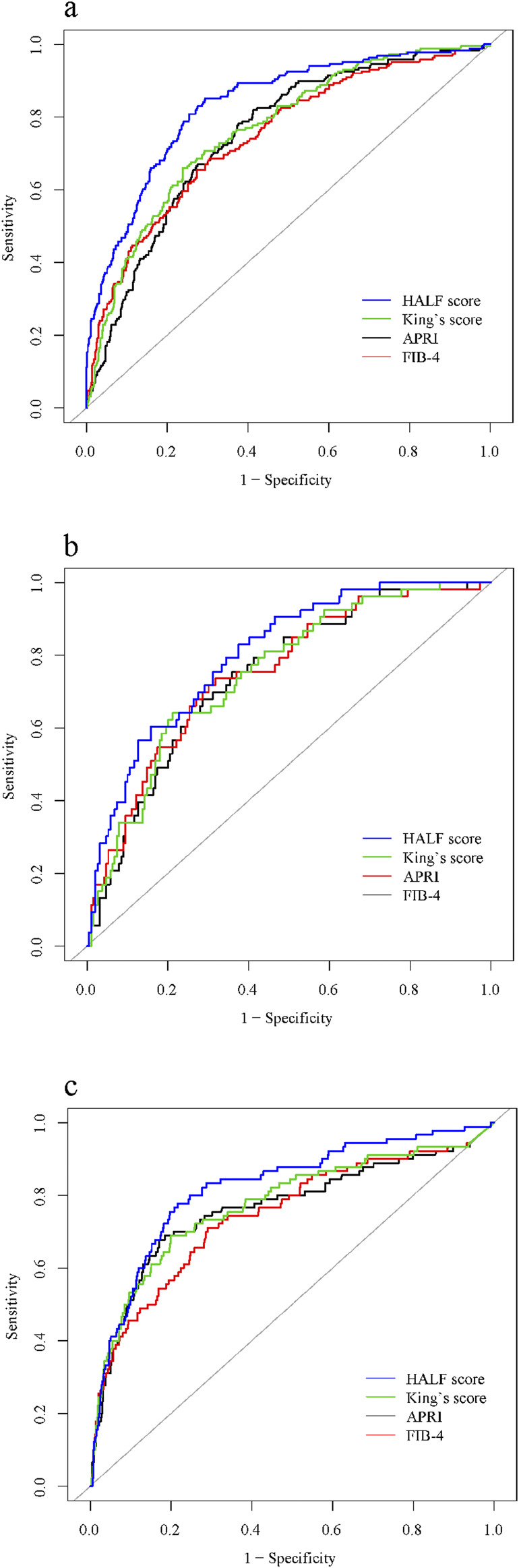
Area under receiver operating characteristic (ROC) comparison of HALF index, APRI, FIB-4, and King’s score. **(A)** Training cohort, **(B)** Internal validation cohort, **(C)** External validation cohort. HALF index, HBV-related advanced liver fibrosis index; APRI, Aspartate aminotransferase to platelet ratio; FIB-4, Fibrosis-4 score.

In the training cohort, we constructed the ROC curve of HALF index for diagnosing advanced liver fibrosis and compared it with FIB-4, APRI, and King’s score. The results suggested that the HALF index possessed the highest area under the ROC curve (AUC) (0.834, 95% CI 0.800–0.868, *p* < 0.05). In the internal validation cohort, the HALF index similarly possessed a higher AUC compared to the FIB-4, APRI, and King’s score (0.804, 95% CI 0.742–0.866, *P* < 0.05). In the external validation cohort, we obtained similar results, with the AUC of the HALF index for the diagnosis of advanced liver fibrosis being (0.821, 95% CI 0.771–0.870, *P* < 0.05). More details of the performance of HALF index versus other noninvasive diagnostic scores for the assessment of advanced liver fibrosis in the three cohorts were shown in Figure 5 and Table 3.

**TABLE 3:** Performance of non-invasive models for diagnosing HBV-related advanced liver fibrosis.

Index	AUC	95% CI	Sensitivity	Specificity	NPV	PPV	Accuracy	Cut-off
Train cohort								
HALF index	0.834	0.800–0.868	0.851	0.706	0.932	0.500	0.743	−1.372
APRI	0.750	0.712–0.789	0.819	0.586	0.904	0.406	0.646	0.551
FIB-4	0.747	0.706–0.788	0.686	0.695	0.865	0.488	0.693	1.452
King’s score	0.765	0.726–0.803	0.660	0.761	0.932	0.500	0.735	13.004
Internal validation cohort								
HALF index	0.804	0.742–0.866	0.830	0.624	0.929	0.383	0.669	−1.371
APRI	0.744	0.673–0.815	0.755	0.640	0.903	0.370	0.665	0.640
FIB-4	0.752	0.680–0.825	0.736	0.683	0.902	0.394	0.694	1.435
King’s score	0.752	0.682–0.822	0.642	0.788	0.887	0.460	0.756	14.616
External validation cohort								
HALF index	0.821	0.771–0.870	0.778	0.783	0.964	0.321	0.782	−1.245
APRI	0.763	0.670–0.826	0.678	0.830	0.951	0.345	0.812	0.640
FIB-4	0.745	0.684–0.807	0.711	0.709	0.949	0.244	0.710	1.562
King’s score	0.774	0.714–0.834	0.689	0.800	0.951	0.313	0.787	10.383

HALF index, HBV-related advanced liver fibrosis index; APRI, aspartate aminotransferase to platelet ratio; FIB-4, Fibrosis-4 score; AUC, area under curve; NPV, negative predictive value; PPV, positive predictive value.

### The value of the clinical application of the constructed nomogram

**FIGURE 6:**
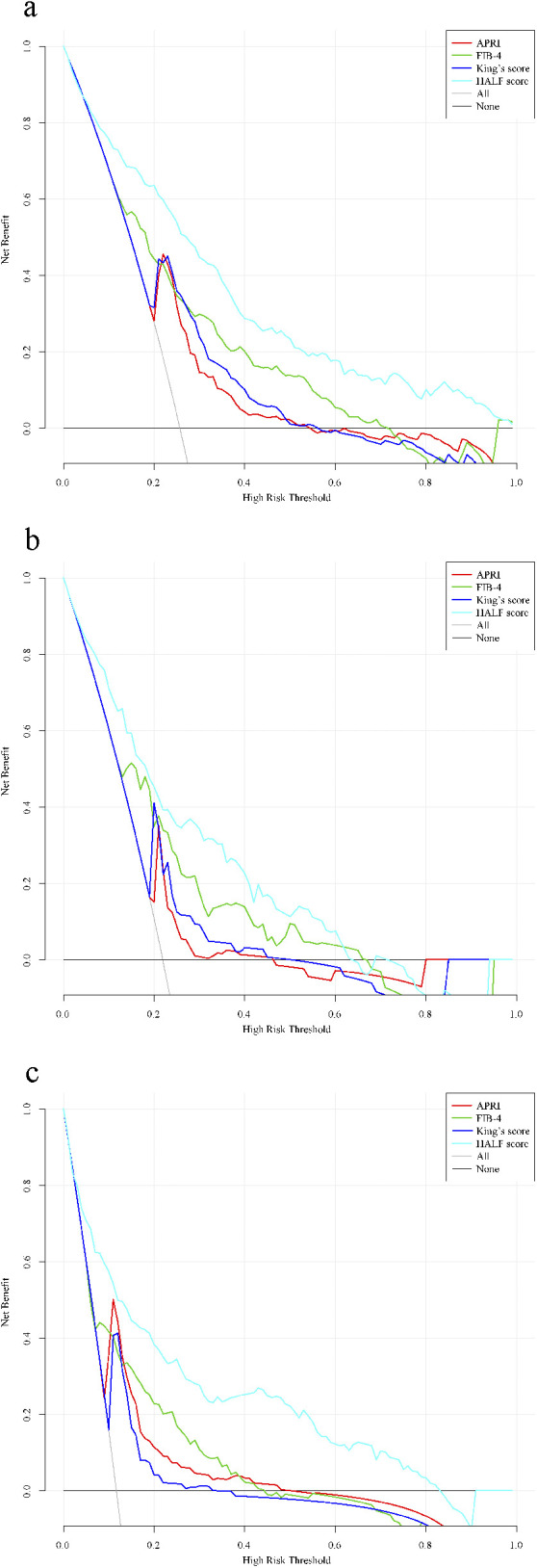
Decision curve analysis for the nomogram. Black line = net benefit when no patient will experience the event; gray line = net benefit when all patients will experience the event. The preferred markers is the marker with the highest net benefit at any given threshold. **(A)** Training cohort, **(B)** Internal validation cohort, **(C)** External validation cohort. HALF index, HBV-related advanced liver fibrosis index; APRI, Aspartate aminotransferase to platelet ratio; FIB-4, Fibrosis-4 score.

Finally, to assess whether the nomogram based on the HALF index could benefit patients in clinical applications, we further constructed decision curves and performed visual comparisons. Overall, in all cohorts, the HALF index predicted the risk of advanced liver fibrosis was able to benefit more patients with a wider range of threshold probabilities than other noninvasive diagnostic scores. Such results were more significant in the training set and external validation cohorts (Figure 6).

## Discussion

CHB is a great challenge to global health and its main mechanism is necrosis and inflammation of liver cells due to the immune response induced by hepatitis B virus. Moreover, due to the presence of covalently closed circular DNA (cccDNA), which makes it difficult to eradicate the virus completely, and most of the patients are at risk of disease progression at any time, including liver fibrosis (Revill and Yuan, 2013). In addition, it is estimated that 60%–70% of the progression of liver fibrosis to cirrhosis is asymptomatic and unsuspected until the onset of complications (Zhang et al., 2021). It would be beneficial to target management of CHB patients at any stage of the disease (Lai and Yuen, 2007). Therefore, it is clinically important to be able to recognize the stage of liver fibrosis in patients with CHB in a timely manner, especially with the use of noninvasive diagnostic scores. In this study, we constructed a nomogram prediction model capable of predicting advanced liver fibrosis in patients with CHB using clinically accessible information. After validation in both internal and external cohorts, the currently constructed model is valid for CHB-associated advanced liver fibrosis and has convenience in clinical practice.

Firstly, we screened several significant predictors by Lasso regression analysis. Among the results of multivariate logistic regression analysis, we found that male patients had a higher risk of advanced liver fibrosis, which may be related to the fact that males have a higher prevalence of CHB. For example, in the cohort we used, the proportion of male patients with CHB was approximately 2–3 times higher than that of female patients, which was similar to the results of previous epidemiologic investigations (Chien et al., 2023). Actually, the existence of sex differences in chronic liver disease associated with viral hepatitis B has been noted early on, and researchers have suggested that this is related to sex hormone differences and not to gender *per se* or environmental factors (Wang et al., 2015). Similar to the presence of gender differences in CHB, hepatitis B virus (HBV)-associated hepatocellular carcinoma (HCC) is more common in males as well as in postmenopausal females. This is related to the ability of androgens to positively regulate HBV virus replication, which was confirmed in experiments with castrated mice (Wang et al., 2009). In laboratory tests, our results indicated that ALB and GLB were important predictors for the diagnosis of CHB-associated advanced liver fibrosis. ALB is mainly produced by hepatocytes, and the decrease of ALB reflects the poor basal nutritional status of patients with CHB-related liver fibrosis. In addition, in a recent study, researchers found that ALB positively regulates the expression of nuclear receptor-associated protein 1 in the liver, thereby inhibiting hepatic stellate cell epithelial mesenchymal transition and extracellular matrix deposition in order to exert antifibrotic effects (Song et al., 2024). GLB is mainly produced by immune cells, and relevant studies have shown that higher levels of GLB not only reflect the level of immunity, but also the degree of hepatic inflammation, which is especially pronounced in patients with CHB-related liver fibrosis (Zheng et al., 2010). GGT, which is mainly derived from hepatocytes, is a membrane-bound enzyme and is an important marker in response to hepatocyte injury (Everhart and Wright, 2013) and previous studies have demonstrated that GGT may have a better predictive value for significant liver fibrosis or even cirrhosis compared to ALT and AST alone (Yu et al., 2016), which is the same as the results of the present study. Similar to GGT, AKP is also a marker of liver fibrosis and is a more stable parameter compared to other liver biochemical parameters (Pan et al., 2022). PT represents the synthetic function of hepatocytes and is closely associated with severe liver inflammation and fibrosis progression (Li et al., 2005). It is associated with decreased coagulation factors and lack of fibrinogen synthesis, suggesting that the synthetic capacity of hepatocytes decreases with increasing liver fibrosis (Fung et al., 2010). Hypersplenism during the progression of liver fibrosis is one of the reasons for the decrease in PLT, besides, the decrease in PLT-stimulating factor due to the elevation of PLT-associated immunoglobulin is also the main reason (Gallo et al., 2022). In any case, PLT is an important indicator of the functional status of the liver in patients with CHB and liver fibrosis, and the ability to establish a prediction model for liver fibrosis using PLT as a predictor has been unanimously recognized by researchers (Zhang et al., 2021; Wang H. et al., 2023; Xu et al., 2020). In summary, the predictor variables screened in this study were valid and they were utilized to construct a nomogram prediction model capable of diagnosing CHB-related advanced liver fibrosis.

In our study, a nomogram prediction model called HALF index was established to be effective in diagnosing CHB-associated advanced liver fibrosis and validated in both internal and external validation cohorts. However, our study still had several limitations. First, this study was based on retrospective clinical data, which is currently not confirmed by prospective cohort studies, and thus requires rigor in interpreting our results and validation in cohorts from other centers. Second, although liver biopsy information was collected from all patients in all cohorts of this study, liver biopsy examination has become increasingly difficult to accept in patients with CHB primaries with advances in imaging technology, which has led to limitations in follow-up studies based on this study. Third, it is important to recognize that the sample size of patients who were adjudicated as S4 in our study was limited, which was more evident in the internal validation cohort. Therefore, our model was not validated in the diagnosis of cirrhosis. Fourth, we assessed the stage of liver fibrosis for the patients in this study with the Scheuer scoring system, so the current results might not be representative of patients with advanced liver fibrosis as adjudicated by other scoring systems. Fifth, in this study, Lasso regression analysis has a particular advantage in screening potential predictor variables. However, controlling the number of selected variables by adjusting the λ parameter implies that the larger the λ parameter the more predictor variables are excluded, and such a treatment may risk underfitting. Finally, due to the lack of certain available data in the included cohorts, we regret that we did not compare the diagnostic validity between the HALF index and certain imaging tests (e.g., LUTE) for advanced liver fibrosis.

## Conclusion

In conclusion, the results of this study showed that gender, ALB, GLB, PLT, AKP, GGT and PT were independent predictors for CHB-related advanced liver fibrosis. The nomogram model based on these parameters was effective in predicting the probability of CHB-associated advanced liver fibrosis and has excellent clinical utility.

## Data Availability

The raw data supporting the conclusions of this article will be made available by the authors, without undue reservation.
