# Supplementary material for: Development and validation of a nomogram for predicting advanced liver fibrosis in patients with chronic hepatitis B
Source: Front Mol Biosci. 2024 Sep 2;11:1452841. doi: 10.3389/fmolb.2024.1452841 (PMC11403247; doi:10.3389/fmolb.2024.1452841)
Supplement: Supplementary file 1 [file Table1.DOC]

Supplementary table 1. Missing patterns and frequency of Variables

| Variables | Frequency | Variables | Frequency |
| --- | --- | --- | --- |
| Nothing | 662 | AFP | 75 |
| AKP | 4 | log _10_ (HBV DNA) | 27 |
| APTT | 2 | Tbil | 1 |
| TC, TG | 131 | AFP, log _10_ (HBV DNA) | 11 |
| AFP, APTT | 1 | NEU, LYC | 8 |
| Ibil, AKP | 2 | AFP, TC, TG | 20 |
| APTT, NEU, LYC | 3 | log _10_ (HBV DNA), TC, TG | 6 |
| TC, TG, APTT | 1 | AKP, TC, TG | 3 |
| AFP, NEU, LYC | 2 | AFP, APTT, NEU, LYC | 5 |
| GLb, Ibil, TC, TG | 1 | Ibil, AKP, TC, TG | 1 |
| AFP, TC, TG, APTT | 1 | AFP, log _10_ (HBV DNA), TC, TG | 1 |
| AFP, Ibil, AKP, TC, TG | 3 | AFP, TC, TG, APTT, NEU, LYC | 2 |
| Alb, Glb, Tbil, Ibil, TC, TG | 1 |  |  |

AFP, Alpha fetoprotein; AKP, Alkaline phosphatase; HBV DNA, Hepatitis B virus deoxyribonucleic acid; APTT, Activated partial thromboplastin time; Tbil, Total bilirubin; TC, Total cholesterol; TG, Triglyceride; NEU, Neutrophil count; LYC, Lymphocyte count; Ibil, Indirect bilirubin; GLb, Globulin; Alb, Albumin.

Supplementary table 2. Clinical characteristics of studied patients in the training cohort and external validation cohort

| Characteristics | Training cohort | External validation cohort | P value |
| --- | --- | --- | --- |
| Sample size | 732 | 771 |  |
| Age (years) | 37.000 (29.00-45.000) | 43.000 (34.000-51.000) | <0.001 |
| Gender |  |  | <0.001 |
| Male | 74.727 | 63.813 |  |
| Female | 25.273 | 36.187 |  |
| AFP (ng/ml) (%) |  |  | 0.008 |
| < 20 | 80.465 | 83.528 |  |
| ≥ 20 | 7.240 | 3.632 |  |
| Unclear | 12.295 | 12.840 |  |
| log _10_ (HBV DNA) | 5.530 (3.868-7.250) | 4.000 (3.000-6.000) | <0.001 |
| Alb (g/L) | 41.050 (37.675-44.000) | 42.200 (39.600-45.000) | <0.001 |
| Glb (g/L) | 26.500 (23.500-29.600) | 25.800 (22.700-29.400) | 0.020 |
| Tbil (umol/L) | 13.600 (10.300-18.400) | 11.900 (8.800-16.600) | <0.001 |
| Ibil (umol/L) | 10.300 (8.100-14.700) | 8.350 (5.500-12.900) | <0.001 |
| ALT (u/l) | 47.000 (27.000-84.000) | 29.0000 (18.000-47.000) | <0.001 |
| AST (u/L) | 34.000 (24.000-52.250) | 25.000 (20.000-34.000) | <0.001 |
| AKP (u/L) | 76.000 (61.000-98.000) | 73.000 (61.000-91.000) | 0.031 |
| GGT (u/L) | 26.000 (17.000-50.000) | 20.000 (13.000-34.000) | <0.001 |
| TC (mmol/L) (%) |  |  | <0.001 |
| < 5.2 | 73.634 | 62.905 |  |
| ≥ 5.2 | 8.060 | 7.263 |  |
| Unclear | 18.306 | 29.832 |  |
| TG (mmol/L) (%) |  |  | <0.001 |
| < 1.7 | 69.809 | 60.311 |  |
| ≥ 1.7 | 11.885 | 9.598 |  |
| Unclear | 18.306 | 30.091 |  |
| WBC (10^9^/L) | 5.1650(4.210-6.333) | 5.150 (4.320-6.285) | 0.646 |
| NEU (10^9^/L) | 2.725 (2.100-3.410) | 2.875 (2.200-3.620) | 0.007 |
| LYC (10^9^/L) | 1.795 (1.426-2.220) | 1.780 (1.420-2.200) | 0.566 |
| RBC (10^12^/L) | 4.600 (4.200-4.910) | 4.480 (4.080-4.850) | 0.641 |
| Hb (g/L) | 143.000 (130.000-152.000) | 137.000 (124.000-148.000) | <0.001 |
| PLT (10^9^/L) | 158.000 (116.000-198.000) | 162.000 (128.000-194.000) | 0.090 |
| PT (s) | 11.900 (11.100-13.000) | 12.700 (12.200-13.450) | <0.001 |
| APTT (s) | 31.200 (27.700-35.100) | 36.600 (33.725-39.400) | <0.001 |
| INR | 1.030 (0.970-1.1100) | 1.000 (0.940-1.090) | <0.001 |
| Liver fibrosis stage (%) |  |  |  |
| S0 | 3.142 | 2.464 |  |
| S1 | 49.044 | 34.890 |  |
| S2 | 22.131 | 43.32 |  |
| S3 | 12.978 | 7.393 |  |
| S4 | 12.705 | 11.933 |  |
| FIB-4 | 1.212 (0.781-1.993) | 1.203 (0.730-1.810) | 0.111 |
| APRI | 0.570 (0.357-1.039) | 0.363 (0.258-0.597) | <0.001 |
| King’s score | 8.552 (4.802-17.0689) | 6.181 (3.912-10.504) | <0.001 |

AFP, Alpha fetoprotein; AKP, Alkaline phosphatase; HBV DNA, Hepatitis B virus deoxyribonucleic acid; APTT, Activated partial thromboplastin time; Tbil, Total bilirubin; TC, Total cholesterol; TG, Triglyceride; NEU, Neutrophil count; LYC, Lymphocyte count; Ibil, Indirect bilirubin; GLb, Globulin; Alb, Albumin; ALT, Alanine aminotransferase; AST, Aspartate aminotransferase; GGT, glutamyl transpeptidase; WBC, White blood cell count; RBC, Red blood cell count; Hb, hemoglobin; Platelet count; PT, Prothrombin time; INR, International normalized ratio; FIB-4, Fibrosis-4 score; APRI, Aspartate aminotransferase to platelet ratio.
